# Liquid silicone gel injection leading to primary squamous cell carcinoma of the breast

**DOI:** 10.1002/kjm2.12874

**Published:** 2024-07-29

**Authors:** Hidenobu Takahashi, Yen‐Shuo Huang, Chee‐Yin Chai, Jung‐Yu Kan

**Affiliations:** ^1^ Division of Breast Oncology and Surgery, Department of Surgery Kaohsiung Medical University Hospital Kaohsiung Taiwan; ^2^ Department of Pathology Kaohsiung Medical University Hospital Kaohsiung Taiwan; ^3^ Department of Pathology College of Medicine, Kaohsiung Medical University Kaohsiung Taiwan; ^4^ Graduate Institute of Medicine, College of Medicine, Kaohsiung Medical University Kaohsiung Taiwan; ^5^ Institute of Biomedical Sciences, National Sun Yat‐Sen University Kaohsiung Taiwan; ^6^ Taiwan Breast Cancer Society Taipei Taiwan; ^7^ Department of Post Baccalaureate Medicine College of Medicine, Kaohsiung Medical University Kaohsiung Taiwan; ^8^ Kaohsiung Breast Cancer Prevention and Education Society Kaohsiung Taiwan

Primary squamous cell carcinoma (SqCC) of the breast, which represents <0.1% of all breast cancers, is rare and diagnostically challenging, possibly having high mortality due to its large size, distant metastasis, and rapid progression. Bagged silicone possibly induces primary breast SqCC. Liquid silicone gel was replaced with bagged silicone breast implants; however, this was warned about by the FDA on March 8, 2023.

This case details primary SqCC of the breast in an 81‐year‐old postmenopausal Asian woman with a history of bilateral liquid silicone gel injections in her 20s. She exhibited local heat, swelling, and mild erythema in the left breast. Chest computed tomography (CT) showed a 10.3 cm left breast tumor with lymph node and bilateral lung metastases, significant calcifications around the silicone injection sites, and pathological findings of squamous cell carcinoma with keratinization. (Figure 1) Tumor markers revealed elevated serum SqCC antigen at 28.0 ng/mL, with normal CEA and CA15‐3 levels. Treatment included a left modified radical mastectomy and sentinel lymph node biopsy, with negative estrogen receptor, progesterone receptor, human epidermal growth factor receptor 2, and GATA Binding Protein 3, and a Ki‐67 index of 15%.

**FIGURE 1:**
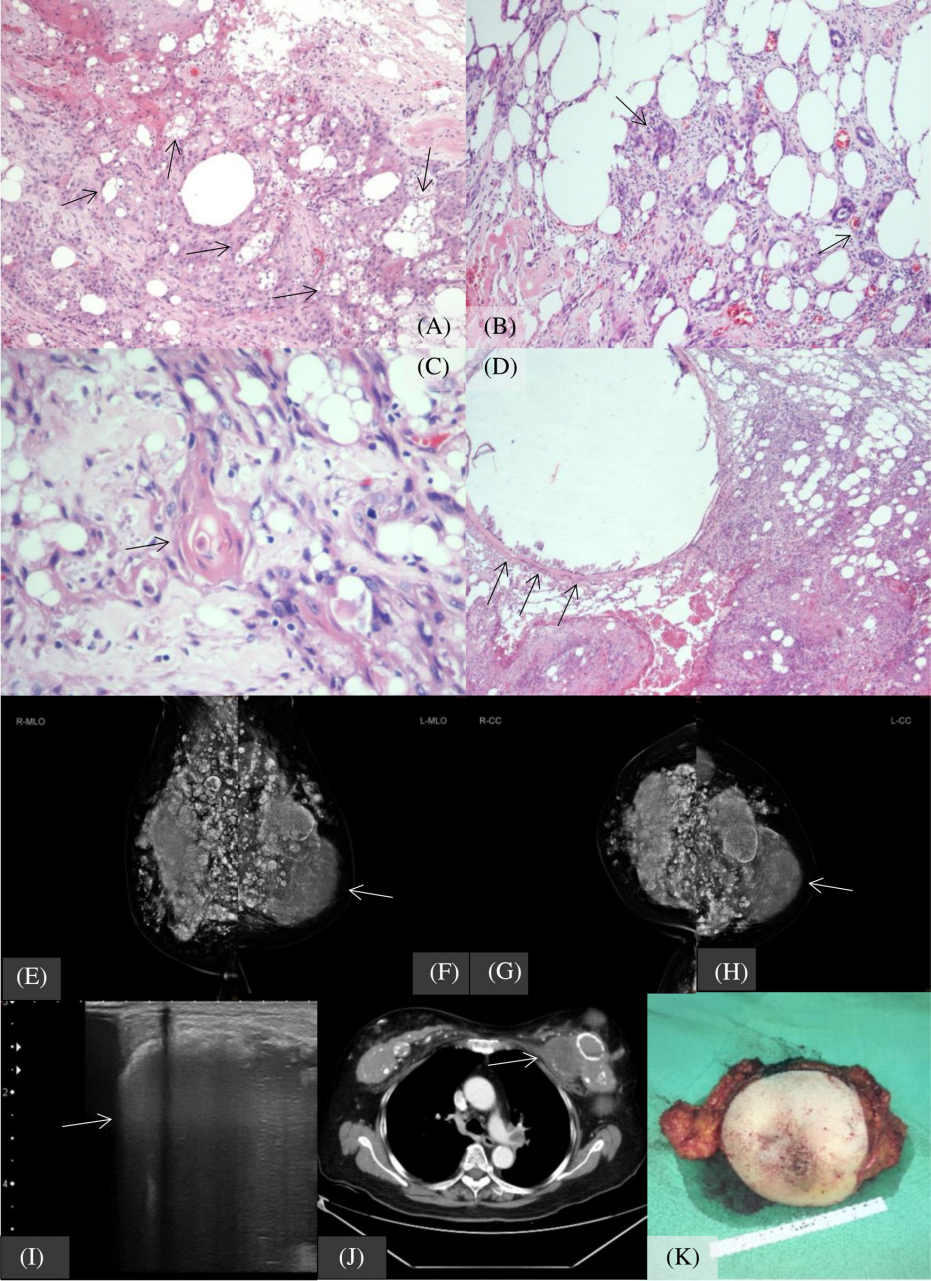
(A) (H&E; original magnification, 100×) Section shows invasive squamoid cells infiltrated into the mammary tissue with abundant surrounding vacuolated cavities and empty spaces. (B) (H&E; original magnification, 200×) Presence of foreign body reaction with multinucleated giant cells and infiltration of inflammatory cells are noted. (C) (H&E; original magnification, 400×) Higher magnification showing atypical squamoid cells with prominent keratinization. (H&E; original magnification, 400×). (D). (H&E; original magnification, 40×) Large cavities adjacent to the squamous cell carcinoma with calcification is identified. (E–H) (Series of mammography; (E) right MLO view; (F) left MLO view; (G) right CC view; (H) left CC view.) Silicone and/or paraffine injection mastopathy in both breasts. >4 cm nodule in the left breast. BIRADS category 5: Highly suggestive of malignancy. (I) (Breast sonography) Bilateral breast foreign body injection with calcifications and left breast tumor. BIRADs cat 0: Need additional imaging evaluation. (J) (Chest computed tomography with enhancement) Suspect left breast cancer with lymph node metastasis. (K) (Left mastectomy specimen) 13.0 * 10.0 * 5.5 cm in size. Hard elastic breast specimen, grossly no skin inflammation and ulceration.

Primary SqCC of the breast, representing less than 0.1% of all breast cancers, is rare and diagnostically challenging. The 2003 WHO classification mandates three criteria for diagnosing breast SqCC: (1) Exclusive presence of SqCC without other neoplastic changes, (2) Absence of SqCC at any other primary site, and (3) No overlying skin or nipple involvement.

Literature indicates that early‐stage breast SqCC typically has a favorable prognosis, yet lacks specific diagnostic and management guidelines.^1^ These tumors are often hormone and HER2 receptor negative, as seen in our case.^2^ Our case, with the largest reported primary breast SqCC (10.3 cm), underscores the lack of standardized treatment protocols. Despite the absence of formal guidelines, our patient underwent surgical treatment without subsequent chemotherapy, radiation, or hormone therapy. However, Aparicio et al. reported no survival advantage for SqCC patients receiving neoadjuvant or adjuvant chemotherapy, in comparison to those who did not receive chemotherapy, highlighting the need for tailored treatment strategies.^3^

However, silicone gel injections, once popular in Taiwan, have been associated with complications like mastitis, sarcoma, and systemic issues such as autoimmune/inflammatory syndrome induced by adjuvants (ASIA) also called human adjuvant disease (HAD) indicate liquid silicone dissemination may lead chronic inflammatory status due to silicone spread.^4^ Our case reveals long‐term risks including significant calcification and chronic inflammation surrounding the injected silicone gel parcels on CT scans, which induces squamous cell metaplasia in future (Figure 1). This case supports the notion that breast SqCC can occur following silicone injections. Microscopically, the breast tissue was infiltrated by invasive carcinoma with squamous differentiation, exhibiting varying degrees of nuclear atypia and prominent keratinization. These neoplastic cells were interspersed with numerous vacuolated cavities and empty spaces of different sizes. In the surrounding mammary tissue, an exuberant foreign body reaction was observed, characterized by the presence of multinucleated giant cells and lymphocytic infiltration. Based on the patient's clinical history and the morphological findings, it is suggested that the occurrence of squamous cell carcinoma in the breast is associated with the silicone injection (Figure 1).^5^

This report highlights a case of SqCC linked to silicone gel injections and the largest SqCC currently reported; however, the latent population numbers who received silicone gel injection was unclear, when the injection was popular in Taiwan, the part of population may had received silicone gel injection by non‐authorized medical professional, thus not officially recorded. Owing to the benign appearance on mammograms and ultrasounds, individuals who received silicone gel injections may require additional screening methods such as SqCC tumor marker assessment and magnetic resonance imaging/CT studies and guided biopsy to suspicious lesion.

In conclusion, this case of primary breast SqCC following silicone gel injection emphasizes the urgent need for research on the link between silicone gel, implants, and SqCC development in the breast. This highlights the carcinogenic risk associated with liquid silicone gel injections, as evidenced by the high occurrence of SqCC. This case report responds to a recent FDA warning and emphasizes the need for heightened awareness and screening of individuals with past silicone gel injections.

## CONFLICT OF INTEREST STATEMENT

All authors declare no conflict of interest.
